# Feedback Loops Shape Oxidative and Immune Interactions in Hepatic Ischemia–Reperfusion Injury

**DOI:** 10.3390/antiox14080944

**Published:** 2025-07-31

**Authors:** Kenneth J. Dery, Richard Chiu, Aanchal Kasargod, Jerzy W. Kupiec-Weglinski

**Affiliations:** Division of Liver and Pancreas Transplantation, Department of Surgery, The Dumont-UCLA Transplantation Center, David Geffen School of Medicine at UCLA, Los Angeles, CA 90095, USA; rchiu2003@g.ucla.edu (R.C.); akasargod@g.ucla.edu (A.K.); jkupiec@mednet.ucla.edu (J.W.K.-W.)

**Keywords:** hepatocytes, cross-talk, inflammation, ischemia–reperfusion injury, Kupffer cells, organ transplantation, oxidative stress, ROS

## Abstract

Reactive oxygen species (ROS) play a dual role as both essential signaling molecules and harmful mediators of damage. Imbalances in the redox state of the liver can overwhelm antioxidant defenses and promote mitochondrial dysfunction, oxidative damage, and inflammation. Complex feedback loops between ROS and immune signaling pathways are a hallmark of pathological liver conditions, such as hepatic ischemia–reperfusion injury (IRI). This is a major cause of liver transplant failure and is of increasing significance due to the increased use of marginally discarded livers for transplantation. This review outlines the major enzymatic and metabolic sources of ROS in hepatic IRI, including mitochondrial reverse electron transport, NADPH oxidases, cytochrome P450 enzymes, and endoplasmic reticulum stress. Hepatocyte injury activates redox feedback loops that initiate immune cascades through DAMP release, toll-like receptor signaling, and cytokine production. Emerging regulatory mechanisms, such as succinate accumulation and cytosolic calcium–CAMKII signaling, further shape oxidative dynamics. Pharmacological therapies and the use of antioxidant and immunomodulatory approaches, including nanoparticles and redox-sensitive therapeutics, are discussed as protective strategies. A deeper understanding of how redox and immune feedback loops interact is an exciting and active area of research that warrants further clinical investigation.

## 1. Introduction

**Figure 1:**
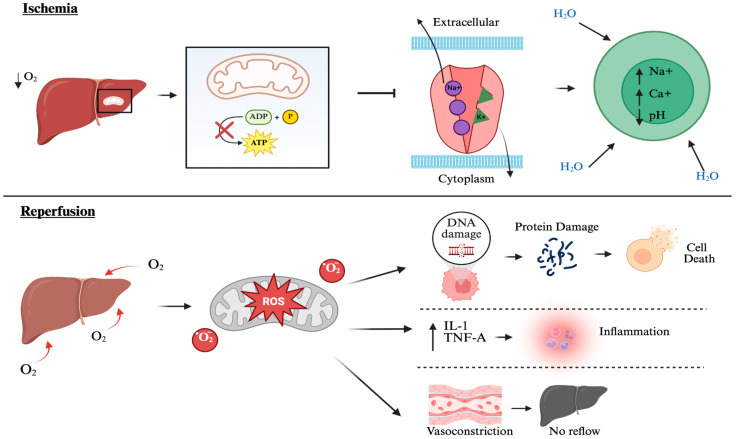
Mechanism of hepatic IRI. Liver injury begins with the oxygen-depleted ischemic phase, where a lack of blood flow to the liver creates an oxygen-poor environment. This environment then inhibits mitochondrial production of ATP, which in turn inhibits the Na^+^/K^+^ ATPase pump, leading to an increased ion concentration in the cells and swelling. This sets the stage for the reperfusion phase, where oxygen is reintroduced to the liver, but is turned into oxygen free radicals. ROS have multiple damaging effects, including DNA and protein degradation, leading to cell death, increased inflammation, and vasoconstriction, preventing the reflow of blood back into the liver. Created by Biorender.com.

In the past, liver research focused mainly on elucidating how the dysregulation of the metabolic and detoxifying functions of hepatocytes contributes to systemic disease [1]. Some well-researched liver pathologies include ischemia–reperfusion injury (IRI), metabolic syndrome, nonalcoholic fatty liver disease (NAFLD), viral hepatitis, and hepatocellular carcinoma [2]. Hepatic IRI occurs during organ transplantation when donor tissues are exposed to a period of ischemia or lack of oxygen, initiating a cascade of events upon reperfusion that includes the release of pro-inflammatory cytokines [3] (Figure 1). Initially, mitochondrial dysfunction leads to the rapid generation of reactive oxygen species (ROS), which leads to oxidative stress, tissue inflammation, and cell death, contributing significantly to graft dysfunction and poor transplant outcomes [4,5]. Despite diverse etiologies, these liver conditions are unified by a common pathogenic feature: the overproduction of ROS, which drives oxidative stress and cellular dysfunction [2].

ROS are highly reactive molecules generated as the byproducts of normal metabolism or induced by inflammatory stimuli [6]. They are generated through multiple cellular pathways, and their detrimental effects have been extensively documented [7]. During oxidative stress, especially under conditions such as IRI, enzymatic pathways, including xanthine oxidase and NADPH oxidase, generate excessive levels of ROS, which exacerbate cellular damage and contribute to mitochondrial dysfunction [8].

**Figure 2:**
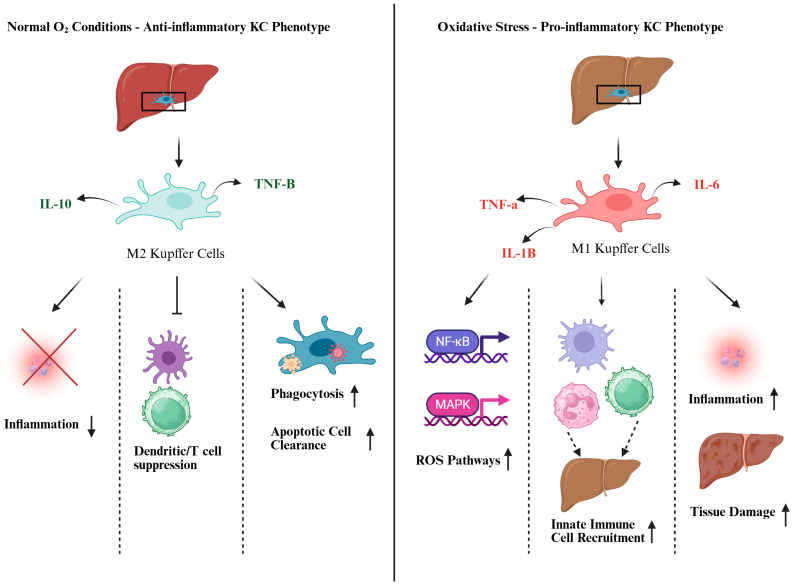
Kupffer cell phenotype changes during oxidative stress. Under normoxic conditions, Kupffer cells exhibit M2 macrophage qualities, performing typical functions such as releasing anti-inflammatory cytokines like IL-10 and TNF-β, suppressing dendritic cell and T cell activation, and clearing immune debris and pathogens. However, under oxidative stress, Kupffer cells display M1 macrophage qualities, releasing pro-inflammatory cytokines like TNF-α, IL-1β, and IL-6, recruiting innate immune cells to the liver, and causing tissue damage. Arrows indicate possible Kupffer cell differentiation fates and downstream effects under normal oxygen (anti-inflammatory M2 phenotype) and oxidative stress (pro-inflammatory M1 phenotype) conditions. Created by Biorender.com.

Under physiological conditions, low to moderate ROS levels serve as signaling molecules that modulate hepatocyte function, immune cell activation, and tissue remodeling [9]. However, excessive ROS overwhelms antioxidant defenses, leading to oxidative damage, mitochondrial dysfunction, and the activation of inflammatory cascades [10]. For example, ROS production leads to the recruitment of neutrophils to the cellular environment, furthering the damage to hepatocytes [11]. In response to stress, the damaged liver cells release damage-associated molecular patterns (DAMPs), which then activate toll-like receptor (TLR) pathways, expressing myeloid differentiation genes such as MyD88, and eventually neutrophil extracellular traps (NETs) [12]. This signaling cascade exacerbates liver IRI by activating cytokine-releasing Kupffer cells, initiating the release of interleukins, tumor necrosis factor-alpha (TNFA), increasing the sequestration of CD4+ T cells, and promoting apoptosis [13]. Figure 2 details how Kupffer cells undergo phenotypic changes during oxidative stress.

As a leading cause of organ dysfunction and failure, IRI warrants continued investigation into its oxidant mechanisms [14,15]. Despite decades of intense research, the therapeutic options for many of these liver pathologies remain limited to supportive or symptom-based approaches. Although the importance of oxidative stress in liver disease is well recognized, the complexity of redox-regulated hepatocyte–immune interactions remains incompletely understood. Emerging evidence suggests that intrinsic and therapeutically modulated antioxidant mechanisms can disrupt this damaging feedback loop and restore immunological hemostasis and metabolic balance. This review explores the mechanistic foundations of ROS generation and antioxidant defenses in the liver, specifically focusing on efforts to mitigate IRI but also on understanding how redox dynamics govern hepatocyte–immune cell crosstalk in health and disease.

## 2. Oxidant Mechanisms That Contribute to ROS in Hepatic IRI

### 2.1. Major Sources of ROS

ROS produced by mitochondria can benefit the cellular environment by activating therapeutic antioxidant processes in the event of cellular stress. ROS regulate cell proliferation and differentiation at controlled levels, modulate immune responses, influence autophagy and apoptosis, and act as secondary messengers in signal transduction pathways [16]. Hydrogen peroxide (H_2_O_2_) and superoxide (O_2_^−^**·**) are specific oxidants that function as redox signaling agents. They occur in normally metabolizing cells at a steady-state level and are formed continuously. However, its excess accumulation within hepatocytes can often lead to liver damage [17]. Maintaining cellular homeostasis and overall liver health critically depends on a balanced interplay between oxidative stress mechanisms and antioxidant defense systems [18]. Table 1 highlights some representative studies that focus on oxidative stress-related pathways and their key findings from 2024 to 2025.

**Figure 3:**
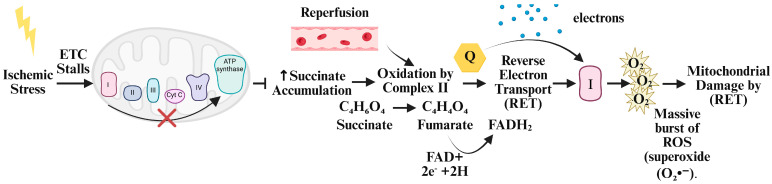
Generation of ROS in the mitochondria from Reverse Electron Transport (RET). Ischemic stress causes stalling in the electron transport chain. Upon reperfusion in the recipient during liver transplantation, mitochondrial succinate levels increase. This causes rapid oxidation to fumarate by succinate dehydrogenase (complex II), driving a surge of electrons into the electron transport chain (ETC). This process, known as reverse electron transport (RET), leads to excessive superoxide (O_2_**·**^−^) production at complex I. RET is triggered by metabolic stress, such as ischemia–reperfusion, and contributes to oxidative damage and redox signaling. Created by Biorender.com.

Recent evidence has underscored the importance of temporal and spatial ROS dynamics as a critical driver of downstream inflammatory and cell death pathways [37]. During ischemic stress, following orthotopic liver transplantation (OLT), mitochondrial metabolism becomes impaired, leading to the accumulation of succinate because of stalled tricarboxylic acid (TCA) cycle activity (Figure 3). The key event occurs during reperfusion; the sudden reintroduction of oxygen enables the rapid oxidation of the accumulated succinate by mitochondrial complex II (succinate dehydrogenase). This process drives electrons into the electron transport chain, creating a highly reduced ubiquinone pool that facilitates reverse electron transport (RET) at complex I [38]. RET is a major source of mitochondrial ROS generation, contributing to oxidative stress, mitochondrial dysfunction, and tissue injury associated with ischemia–reperfusion injury [39].

Studies on succinate show that it is not associated with metabolic syndrome and liver dysfunction, however [40]. One recent study used mice with muscle-specific ablation of succinate dehydrogenase to show that knockout mice demonstrate normal liver histology and oxidative capacity, suggesting that additional regulatory mechanisms beyond succinate accumulation may govern mitochondrial ROS production and tissue damage during IRI. For example, a recent study showed that hepatic mitochondrial fat oxidation, lipolysis, and TCA cycle activity are enhanced independent of mitochondrial calcium levels through cytosolic calcium–CAMKII signaling [41]. The authors used a liver-specific mitochondrial calcium uniporter knockout mouse model (MCU KO) to reduce mitochondrial calcium ([Ca^2+^]mt) while elevating cytosolic calcium ([Ca^2+^]cyt). Using in vivo metabolic flux analysis, radiolabeled [^14^C_16_] palmitate oxidation assays, liver slice studies, and CAMKII modulation (activation and knockdown) showed that increased [Ca^2+^]cyt activates CAMKII, driving enhanced hepatic fat oxidation and reduced triacylglycerol accumulation. Whether elevated [Ca^2+^]cyt-CAMKII activity may promote substrate flux through the TCA cycle to reduce succinate accumulation during ischemia, thereby limiting RET-driven ROS upon reperfusion, remains to be determined.

Another major source of ROS in hepatic IRI comes from the NADPH oxidase family of NOX enzymes, particularly during reperfusion [42]. NOX enzymes transfer electrons from NADPH inside the cell to oxygen-rich membranes, generating H_2_O_2_ and superoxide O_2_^−^ species. During reperfusion, the sudden influx of oxygen supply on toll-like receptors (TLRs) on Kupffer cells, endothelial cells, and hepatocytes upregulate NOX expression and activity. Subsequent ROS production can influence lipid peroxidation, protein oxidation, and DNA damage and act as pro-inflammatory signaling molecules that amplify cytokine production and immune cell recruitment [43]. A recent study strengthened our understanding of how NOX enzymes amplify oxidative stress and inflammation [44]. The authors showed that rapamycin reduction in NOX enzymes depressed pro-inflammatory cytokine levels and apoptotic markers in a murine fibrotic liver IRI model. Cytochrome P450 is another source of ROS, particularly in monooxygenases that catalyze the oxidation of various substrates (drugs, toxins, fatty acids) using molecular oxygen (O_2_) and NADPH [45]. In a recent study, the authors showed that iridoid glycoside aucubin, a natural plant-derived compound from *Plantago asiatica*, reduces the ROS in liver IRI by suppressing key inflammatory and oxidative stress pathways. Their key data showed that aucubin inhibited the signaling pathways that involved High Mobility Group Box 1 (HMGB1), TLR-4, and Nuclear Factor kappa-light-chain-enhancer of activated B cells (NF-κB) [46]. Moreover, aucubin reduced mitochondrial dysfunction, apoptosis, and Cytochrome P450 expression, which are all directly associated with lowering ROS levels and protecting hepatic tissue.

Finally, recent studies have focused on the unfolded protein response as a mechanism of generating ROS [47]. During cellular stress, such as oxygen deprivation during the ischemic IRI phase, ATP levels drop, and protein folding halts. This causes unfolded protein accumulation in the endoplasmic reticulum (ER) and hyperactivates enzymes such as Ero1 and protein disulfide isomerase, leading to excess H_2_O_2_ formation. Indeed, a recent study showed that hypothermic oxygenated perfusion (HOPE) effectively mitigated ER stress by modulating unfolded protein response (UPR) signaling [47]. The authors showed that HOPE activated the Janus kinase 2 (JAK2)/ Signal Transducer and Activator of Transcription 3 (STAT3) pathway, leading to the upregulation of the Hematopoietic Cell-Specific Lyn Substrate 1-Associated Protein X-1 (HAX1). This multifunctional protein has been shown to be involved in regulating apoptosis, calcium homeostasis, and cell survival, particularly under stress conditions. These studies show that hepatic ROS arises from metabolic, enzymatic, and signaling disruptions during I/R stress. A thorough understanding of ROS production’s temporal and spatial mechanisms may provide the foundation for identifying strategies to mitigate hepatic injury and improve clinical outcomes.

### 2.2. Key Hepatic and Immune Cell Players

As the principal parenchymal cells of the liver, hepatocytes act as the central regulators of IR-triggered oxidative stress [48]. Not only are they responsible for metabolic homeostasis, detoxification, and protein synthesis, but they also interact with other liver cells, like Kupffer cells and stellate cells, to maintain local homeostasis and responses to injury [49]. Hepatocytes are equipped with endogenous antioxidant systems such as glutathione, superoxide dismutase, and catalase to play a dual role as both sources and targets of ROS [50]. Hepatocytes adapt to rising ROS levels by activating redox-sensitive transcription factors such as nuclear factor erythroid 2–2-related factor 2 (NRF2) [51]. A high oxygen tension initiates a mechanism in hepatocytes that causes NRF2 dissociation from its cytoplasmic inhibitor KEAP1 [52]. This causes its translocation to the nucleus, where it promotes the expression of over 200 genes involved in antioxidant defense, detoxification, and metabolic regulation, including heme oxygenase-1 (HO-1), NAD(P)H quinone dehydrogenase 1 (NQO1), and GSH synthesis enzymes [53,54].

The relationship between mitochondrial ROS-driven inflammation and lipid accumulation in hepatocytes was recently interrogated using primary cultured hepatocytes in a model of NAFLD [55]. Zaluzanin C, a sesquiterpene lactone [56], protected hepatocytes from mitochondrial ROS by preventing Tumor Necrosis Factor Receptor 1 (TNFR1) induction. This study also showed that mitophagy, a specialized type of autophagy that targets the mitochondria [57], induces and upregulates Peroxisome proliferator-activated receptor gamma coactivator 1-alpha (PGC1α), Mitochondrial Transcription Factor A (TFAM), Nrf1, and β-oxidation gene expression, indicating its dual antioxidant and metabolic benefit. In a related investigation, it was demonstrated that hepatocytes respond to LPS-induced oxidative stress by increasing ROS production and cytokine expression, contributing to hepatic inflammation [58]. Rocaglamide-A, a natural bioactive compound extracted from plants of the genus *Aglaia* with known potent pharmacological properties, was shown to be able to reduce ROS levels in hepatocytes by upregulating antioxidant genes and inhibiting the phosphorylation of c-Jun N-terminal kinase (JNK) and Activator Protein-1 (AP-1), key signaling molecules activated by LPS. Rocaglamide-A was able to mitigate nitrosative (e.g., reactive nitrogen species (RNS), such as nitric oxide (NO)) and oxidative stress, highlighting hepatocytes as both sources and targets of ROS-mediated inflammatory signaling in liver injury.

Liver sinusoidal endothelial cells (LSECs) and hepatic stellate cells also play important roles in modulating redox balance and mediating the progression of hepatic IRI [59]. LSECs line the walls of the hepatic sinusoids, which are the specialized capillary-like blood vessels in the liver [60]. During IRI, LSECs experience oxidative stress marked by mitochondrial dysfunction and NADPH oxidase activation, leading to excessive ROS generation [61]. This disrupts the fenestrated architecture of the sinusoids, impairs barrier integrity, and facilitates leukocyte adhesion and transmigration. By comparison, hepatic stellate cells are located in the narrow Disse space between hepatocytes and LSECs [62]. These cells store vitamin A as part of their quiescent (non-activated) state and become activated upon liver injury or I/R stress [63]. Notably, hepatic stellate cells transform upon activation into myofibroblast-like cells that secrete ECM components such as collagen, contributing to fibrosis [64].

Both LSECs and hepatic stellate cells were recently interrogated using single-cell RNA sequencing and transcriptome RNA sequencing in a hepatic model of IRI following transplantation [65]. The clustering analyses revealed 11 distinct cell types across 25 clusters, including 5 non-immune and 6 immune populations. Non-immune cells included hepatocytes, endothelial cells, stellate cells, cholangiocytes [66], and erythroid cells, while immune cells consisted of macrophages, monocytes, NK/T cells [67], B cells [68], and plasma cells [69]. Differential gene expression analysis between pre-perfusion (PP) and post-reperfusion (PR) tissues identified key dysregulated genes in both immune and non-immune liver cell populations. Among the immune-modulating non-parenchymal cells, LSECs showed 14 upregulated and 3 downregulated genes in PR tissue. Several upregulated genes, such as the Heat Shock Protein Family E Member 1 (HSPE1), were associated with the heat shock protein (HSP) signaling pathway. Additional genes, such as BCL2-associated athanogene 3 (BAG3), are implicated in cell cycle control and LSEC homeostasis disruptions. Inflammatory mediators like Annexin A1 (ANXA1) were suggestive of endothelial dysfunction and liver transplant-associated injury. Notably, this study demonstrated a novel function of LSECs in the bidirectional crosstalk between cells of the liver and monocytes. Intercellular communication analysis using CellPhoneDB (Wellcome Sanger Institute, Hinxton, United Kingdom) [70], a database designed to analyze cell–cell communication using single-cell transcriptomic data, revealed the upregulation of the ANXA1-Formyl Peptide Receptor 2 (FPR2) signaling axis. This study used immunofluorescence, flow cytometry, and Western blot analyses to validate their transcriptomic data in a murine IRI model and strengthen their bioinformatics data. These findings point to a mechanism where the LSEC–monocyte ANXA1–FPR2 axis may suggest a feed-forward loop in which LSEC activation enhances monocyte recruitment and inflammatory activity, potentially amplifying liver injury during reperfusion. This study also places this interaction within broader immune regulatory networks, including TLR signaling, NET formation, and Notch1-mediated pathways, which may amplify oxidative stress and immunopathology during IRI.

Besides hepatocytes, LSECs, and hepatic stellate cells, other liver cells, like the Kupffer cells and infiltrating neutrophils, greatly influence hepatocyte metabolism during I/R oxidative stress [71]. Located in the liver sinusoids, Kupffer cells clear cellular debris and harmful substances and detect and remove pathogens. These resident macrophages serve as the first responders to inflammation and injury following activation by DAMPs. Kupffer cells become active during I/R stress, when endogenous molecules like mitochondrial DNA, HMGB1, or ATP, serving as DAMPs, signal tissue damage [72]. Following this, Kupffer cells produce ROS as part of the inflammatory response, leading to sustained liver injury, due to its role as signaling molecules to amplify inflammation by activating transcription factors like NF-κB, promoting the production of pro-inflammatory cytokines (e.g., TNF-α, IL-1β).

Their role in shaping the redox landscape of the hepatic microenvironment is an area of active research [73,74]. For example, a recent study explored how Kupffer cells use Peroxisome Proliferator-Activated Receptor Gamma (PPARγ)-mediated metabolic reprogramming to regulate oxidative stress and inflammation in NAFLD. PPARγ is a nuclear receptor and transcription factor that regulates lipid metabolism, glucose homeostasis, and anti-inflammatory responses [75]. The authors found that PPARγ activation enhanced fatty acid oxidation, reduced ROS production, and suppressed pro-inflammatory cytokine secretion. By linking the antioxidant effect to activation of the Keap1-Nrf2 pathway, the study showed a link between Kupffer cell ROS regulation and hepatocyte inflammatory response [76].

As the most abundant white blood cell type in the bloodstream, neutrophils form an integral line of innate defense following I/R stress. These granulocytic leukocytes rapidly respond to infection or tissue injury by migrating to affected sites. Neutrophils engulf pathogens and debris by phagocytosis, release ROS to kill microbes, and form neutrophil extracellular traps (NETs), web-like structures of DNA and proteins, to trap and neutralize pathogens. Notably, the characterization of NETs in the context of hepatic I/R oxidative stress has recently been an area of active investigation. For example, our group recently showed that neutrophils regulate bioactive lipid sphingosine-1-phosphate (S1P) and its receptors S1PR2 and S1PR3 following hepatic IRI caused by OLT [77]. The S1P–S1PR2/3 signaling axis act in concert as part of a group of G-protein-coupled receptors (GPCRs). Notably, the study identified the long isoform of Carcinoembryonic Antigen–Related Cell Adhesion Molecule 1 (CEACAM1), a transmembrane glycoprotein with extracellular Ig-like domains and variable cytoplasmic tails generated by alternative splicing [78,79,80], as the regulator of S1P–S1PR2/3-dependent NETosis [77]. In another recent study, the authors used microarray, bulk RNA-seq, and single-cell RNA-seq datasets from the Gene Expression Omnibus database [81]. The study’s novelty was that patients stratified by expression levels of NET-related genes exhibited distinct clinical profiles, with high or low expressers correlating with different post-transplant outcomes. Moreover, specific NET-associated gene signatures were predictive biomarkers for early allograft dysfunction following liver transplantation. A recent separate investigation also showed that during hepatic IRI, crosstalk between neutrophils and macrophage-derived Spleen tyrosine kinase (SYK) drives poor postoperative outcomes and tumor recurrence. The administration of pharmacologic SYK inhibitors significantly attenuated tissue damage by reducing neutrophil recruitment, NETs formation, NLRP3 inflammasome assembly, and liver inflammation [82]. These collective studies reveal a dynamic interplay among the native and non-parenchymal cells of the liver in terms of orchestrating the redox balance and immune response during hepatic IRI. Working towards understanding how their intercommunication drives liver injury may provide multiple potential targets for antioxidants and immunomodulatory intervention.

## 3. Immune–Hepatic Crosstalk in Response to Oxidative Stress

### 3.1. Innate Immune Responses

**Figure 4:**
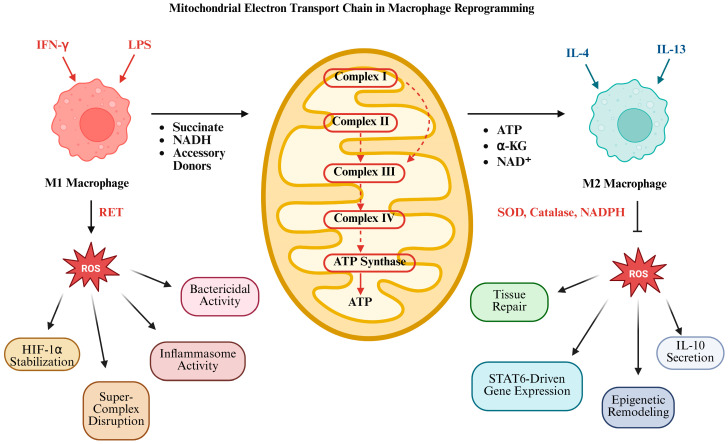
The mitochondrial ETC guides macrophage polarization into pro-inflammatory (M1) and anti-inflammatory (M2) states. In M1 macrophages, stimuli such as LPS and IFN-γ drive glycolysis and succinate accumulation, fueling reverse electron transport (RET) and mitochondrial ROS (mtROS) production. This promotes HIF-1α stabilization, inflammasome activation, and bactericidal activity. M2 macrophages, activated by IL-4/IL-13, rely on oxidative phosphorylation, fatty acid oxidation, and efficient ETC function with minimal ROS. The ETC supplies ATP, α-ketoglutarate, and NAD^+^, supporting IL-10 secretion, tissue repair, and epigenetic remodeling. M2-derived antioxidants such as SOD, catalase, and NADPH further inhibit ROS, reinforcing an anti-inflammatory phenotype. Created in Biorender.com.

The bidirectional communication between parenchymal and immune cells and how it interacts through the duality of amplifying oxidative injury yet initiates repair mechanisms is not well defined [83,84]. Some historical studies have shown that Kupffer cells sense DAMPs during I/R oxidative stress and respond by producing ROS, TNF-α, IL-1β, and chemokines (e.g., CCL2) [85]. These mediators comprise hepatocyte mitochondrial function in a feed-forward loop that polarizes Kupffer cells away from pro-inflammatory (M1-like) and towards reparative (M2-like) phenotypes depending on the state of the redox balance [86] (Figure 4). We recently uncovered divergent functions for T cell immunoglobulin and mucin domain containing 4 (Tim4 signaling) depending on whether its source derives from donor liver or recipient-derived tissue [87]. More specifically, we showed that the disruption of donor Tim4 markedly attenuated IRI, whereas the loss of recipient Tim4 unexpectedly increased the hepatocellular damage, ER stress, disturbed lipid metabolism, and increased pro-inflammatory gene program (e.g., CD36, C/EBP Homologous Protein (CHOP), and IL-10). In the clinical cohort, we reported that high hepatic TIM4 levels in transplant patients correlated with a higher BMI, increased ER stress and apoptotic markers, innate/adaptive immune activation, and higher early allograft dysfunction, suggesting worse graft survival. These findings suggest that targeting donor-derived TIM4 signaling, while preserving recipient TIM4 function, may be a viable strategy to preserve postoperative outcomes in liver transplantation.

### 3.2. Adaptive Immune Involvement

**Figure 5:**
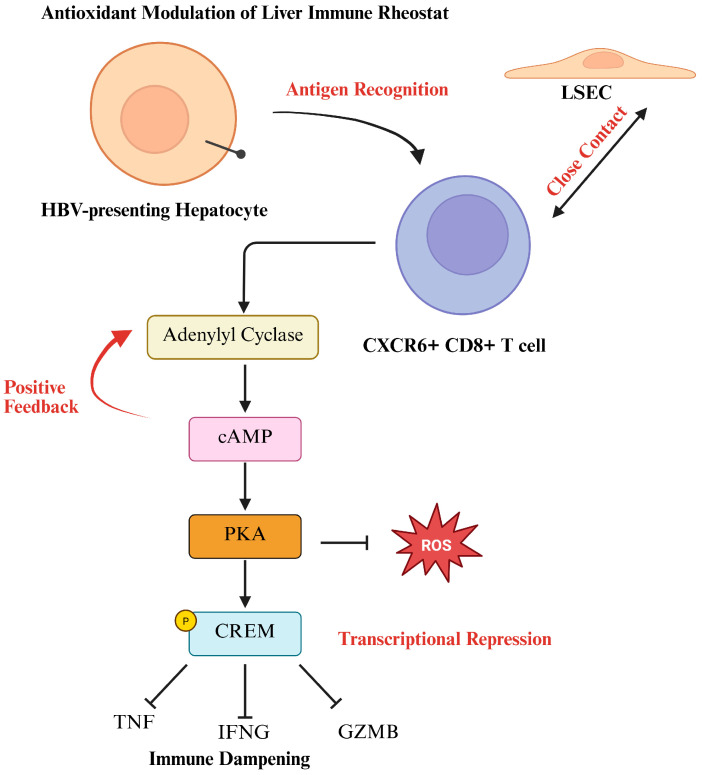
LSEC mediates cAMP Signaling of T cell activity in the liver. The liver maintains immune quiescence through a local rheostat mechanism involving HBV-presenting hepatocytes, liver sinusoidal endothelial cells (LSECs), and CXCR6^+^ CD8^+^ T cells. Antigen recognition leads to LSEC-mediated activation of adenylyl cyclase in T cells, triggering the cAMP–PKA–CREM pathway. This signaling cascade represses effector gene expression (IFNG, GZMB, TNF) and reduces mitochondrial ROS production, collectively dampening T cell activity. This results in a tunable immunosuppressive environment that prevents excessive inflammation and preserves tissue integrity. Created in Biorender.com.

Beyond the innate immune system, the liver regulates adaptive immune responses to hepatic I/R oxidative stress through T cell modulation [87]. In chronic inflammatory conditions such as Hepatitis B virus (HBV) infection, the liver functions as a rheostat, dampening CD8^+^ T cell effector activity to prevent excessive immune-mediated tissue damage [88]. In this study, the authors demonstrated that liver-specific T cells, particularly CXCR6+ CD8 T cells, exhibit signs of exhaustion due to increased cyclic adenosine monophosphate (cAMP)-Protein Kinase A (PKA)-cAMP Response Element Modulator (CREM) signaling, which suppresses T cell activation (Figure 5). While protective in chronic infections, this mechanism may hinder effective immune responses under oxidative stress conditions, impairing the liver’s ability to clear damaged cells and resolve inflammation. In a related recent report, it was demonstrated that hypothermic oxygenated machine perfusion (HOPE) impacts both the oxidative stress response and adaptive alloimmunity, leading to a shift toward immunoregulation [89]. Specifically, HOPE-treated liver grafts showed reduced intrahepatic immune cells and lower activation of ROS pathways compared with static cold storage in 27 patients evaluated in two randomized controlled trials. Mechanistically, perfused livers showed increased circulating and donor-specific regulatory CD4^+^FOXP3^+^CD127^lo^ T cells two weeks post transplant, and decreased CD8^+^ T cell alloreactivity at 3 months. The novelty of this study may implicate pre-transplant conditioning as an active way to modulate the host immune response, offering a new immunological rationale for the clinical benefits of machine perfusion strategies [1].

Building on these observations of the adaptive immune response in hepatic IRI, a recent study showed that monoclonal therapy using anti-TIM1 monoclonal antibodies offered substantial protection against inflammation and apoptosis following administration [68]. The authors attribute the mechanism to the expansion of TIM1^+^ regulatory B cells (Bregs) that increased the secretion of the anti-inflammatory cytokine IL-10. Notably, the protective effects of anti-TIM1 were lost upon B cell depletion with anti-CD20, underscoring the central role of Bregs as a critical immune population in regulating liver inflammation during transplant-related injury.

### 3.3. Feedback Loops Between ROS and Immune Activation

The dysregulation of feedback loops between oxidative stress and immune signaling intensifies the inflammatory cascade during hepatic IRI. Their coordination of complex physiological responses due to ROS build-up is partly responsible for ensuing hepatocyte apoptosis, endothelial dysfunction, and, in severe cases, graft failure following liver transplantation. In the past few years, several representative research studies have addressed the transcriptional regulation, immune signaling, and metabolic stress involved in feedback loops resulting from hepatic IRI. Sun et al. investigated a transcriptional regulatory feedback loop involving Small Nucleolar RNA Host Gene 1 (SNHG1), miR-186-5p, and the transcription factor YY1, which together promoted the survival and proliferation of murine hepatocytes. Notably, the ability of SNHG1 to titrate away the inhibitory regulation of miR-186-5p contributed to a positive feedback loop that ultimately reduced hepatocyte apoptosis [90].

Building on this, a recent study investigated pathological feedback loops where ischemia causes primary cilia shortening in biliary epithelial cells, triggering transcriptional regulation and cellular senescence following liver transplantation [91]. In a related recent report, the authors investigated the positive feedback loop involving murine HMGB1 release, which activates TLR4, leading to NF-κB-driven neutrophil recruitment and further ROS generation, perpetuating liver injury [92]. Mechanistically, the authors show that the active peptide (Ac2-26) of Annexin A1 can be used therapeutically to target feedback loops that lead to neutrophil immune activation, inflammatory cytokine cascades (like TNF-α, IL-1β, IL-6), and ROS amplification. These limited studies highlight how feedback loops confer stability, precision, and adaptability to biological systems. In the future, we will need to understand how multiple feedback loops (hepatocytes -> Kupffer cells, neutrophils -> hepatocytes, T cells -> macrophages) all interact together to mitigate liver damage by modulating both oxidative stress while integrating immune feedback mechanisms.

## 4. Recent Antioxidant Efforts to Mitigate ROS in Hepatic IRI

### 4.1. Targeted Delivery Systems

Nanomedicine has benefited dramatically from the interdisciplinary collaboration of biomedical sciences, physics, and engineering that has led to the design of nanoscale materials that can deliver therapeutic agents with high precision to the liver. New studies using targeted nanoparticles continue to expand our appreciation of how antioxidant strategies can be modulated and tested in preclinical models. Nanoparticles are being designed to target damaged hepatic tissue during IRI, where they can scavenge ROS, modulate immune responses, and release anti-inflammatory drugs [93]. Surface modifications, like Polyethylene glycol (PEG)ylation and ligand targeting, continue to be optimized to enhance controlled-release mechanisms that alter bioavailability while minimizing systemic toxicity [94,95]. PEGylation provides a hydrophilic layer that prevents rapid clearance by the reticuloendothelial system [96], allowing for a longer time in circulation and more particles to accumulate in ischemic liver tissue. This was demonstrated in a recent study by Gao et al. (2025), which used PEGylated catalase (CAT-PEG) to mitigate oxidative stress and inflammation following hepatic IRI [97]. Similarly, a recent study showed how surface modifications using 1,2-distearoyl-sn-glycero-3-phosphoethanolamine (DSPE)-PEG2000-Galactose significantly alleviated a mouse model of hepatic IRI [98]. Specifically, the authors engineered DSPE-PEG2000-Galactose to target mesenchymal stem cell-derived exosomes to the liver. Antioxidant nanoparticles are often modified with targeting ligands such as N-acetylgalactosamine (GalNAc), mannose, and aptamers to improve specificity further. A separate recent investigation found that strategic surface engineering techniques to exploit natural ligand receptor interactions in a model of partial liver warm ischemia [99]. More specifically, mannose receptors (CD206) were coupled to Receptor-Interacting Protein Kinase 3 (RIPK3) siRNA as a part of the delivery system. RIPK3 is a serine/threonine protein kinase critical in regulating necroptosis, whereas mannose receptors are highly expressed on Kupffer cells. In this way, macrophages preferentially took up mannose-decorated nanoparticles via receptor-mediated endocytosis, leading to the knockdown of ROS production in Kupffer cells. Another study demonstrating how surface modifications reduce ROS production in IRI is based on delivering Ceria@Apt, a cerium oxide type of nanoparticles, to the liver [100]. The authors showed that by conjugating cerium oxide to anti-C5a, a natural neutrophil/macrophage chemoattractant that is part of the complement system, nanoparticles could simultaneously scavenge ROS and block C5a-mediated inflammation. While offering a hint to their therapeutic potential, future studies may benefit from determining whether anti-inflammatory nanoparticles used in hepatic IRI would be as versatile for other systemic ischemic disorders.

### 4.2. Enzyme-Based Nano-Therapies

Some recent examples of studies that exploit nanomedicine in hepatic IRI models include one report on the synthesis of ultrasmall cerium (CE)–manganese nanoclusters that exhibit superoxide dismutase (SOD)- and catalase (CAT)-mimetic activity, enabling the efficient scavenging of key ROS including OH˙, O_2_^−^˙, and H_2_O_2_ [101]. The authors showed that cerium–manganese nanoclusters demonstrate strong biocompatibility, preferential liver accumulation, and significant therapeutic efficacy in reducing oxidative stress, hepatocellular injury, and inflammation in a murine model of hepatic IRI. Mechanistically, the dual-valent Ce^3+^/Ce^4+^-dependent redox activity coupled with MnO_2_-mediated catalysis enabled ROS reduction and inflammatory responses by suppressing Kupffer cell and neutrophil activation. The following year, a study showed that cerium oxide (Co) acts as a potent ROS scavenger when administered via laparotomy to rats prior to ischemic/reperfusion stress (I/R) injury [102]. The authors showed that Co therapy leads to lower levels of lipid peroxidation, higher levels of antioxidant enzyme catalase, and detoxification enzyme glutathione S-transferase.

### 4.3. Redox Signaling Modulators Targeting Hepatic IRI Using Nanomedicine

Recent advancements in nanomedicine highlight how redox-sensitive delivery platforms are used to engage metabolic and immune pathways implicated in liver injury. For example, a multifunctional nanodrug, A-MPDA@Fe_3_O_4_@PVP, was recently developed to scavenge ROS to alleviate hepatic ischemia–reperfusion injury [103]. Notably, this nanoplatform combines L-arginine-doped mesoporous polydopamine with ferric oxide and a PVP coating, enabling enzymatic ROS scavenging and immunomodulation. In their preclinical models, A-MPDA@Fe_3_O_4_@PVP lowered oxidative damage and preserved liver function through a mechanism that involved the activation of the PPARγ/NF-κB pathway [103]. Another group developed a ROS-responsive liposomal nanocarrier (RLLs) co-loaded with LY294002 and oridonin to target key signaling pathways in NAFLD. LY294002 is a broad-spectrum inhibitor of phosphoinositide 3-kinases (PI3Ks), while oridonin is a natural Chinese medicinal herb known to have anti-inflammatory, anti-tumor, antioxidant, and anti-fibrotic properties [104,105]. The RLLs were shown to be effective ROS scavengers, could enhance hepatic delivery via Cluster of Differentiation 44 (CD44)-mediated targeting, and stabilize CYP450 activity, leading to improved insulin sensitivity and reduced inflammation and fibrosis in a CCl_4_-induced NAFLD mouse model [95]. CD44 is a transmembrane glycoprotein that functions as a receptor for various ligands, most notably hyaluronic acid, an extracellular matrix component [106]. Cytochrome P450 belongs to the superfamily of heme-containing liver enzymes essential for metabolizing drugs, toxins, and endogenous compounds like hormones and fatty acids [107]. By integrating ROS-responsive materials, immunomodulatory capabilities, and surface modifications like CD44-mediated targeting, nanoparticles may soon offer clinicians the opportunity to deliver drugs actively participating in redox regulation and tissue repair.

Many recent studies have built on the therapeutic potential of flavonoid compounds to mitigate hepatic IRI. For example, hydroxysafflor yellow A (HSYA), a water-soluble flavonoid derived from safflower, was shown to reduce and suppress intracellular ROS and apoptosis. It could also lead to decreased markers of DNA damage and lipid peroxidation, indicating potent antioxidant and cytoprotective effects [108]. Reducing oxidative stress was attributed to lower key inflammatory cytokines such as TNF-α, IL-1β, and IL-6, suggesting that its hepatoprotective function involves direct redox regulation and the modulation of inflammatory signaling pathways. Table 2 shows synthetic antioxidant enhancements to mitigate hepatic IRI.

## 5. Challenges and Future Directions

The grand challenge for the future of liver transplantation translational research lies in bridging the complexity of immune cascades with intricate molecular cross-regulation among diverse hepatic cell populations [114]. Our inability to integrate all the molecular pathways involved in hepatic IRI cascade into a unified framework remains a significant block to therapeutic progress. While these may present limitations for clinical organ transplantation, ample opportunities for insightful and ground-breaking basic research remain. The drive towards improving the donor organ supply represents the most challenging global problem, with the number of patients awaiting life-saving transplant increasing six-fold [114]. Impaired donor tissue quality remains the major contributing factor to the organ shortage. Our aging population, with its pre-existing diseases (e.g., nonalcoholic steatohepatitis, NASH), is particularly susceptible to innate immune-driven tissue damage during cadaver organ harvesting. A stepwise bench-to-bedside translational approach is warranted to promote inflammation-resolving defenses by targeting excessive ROS generation. This will require renewed focus on “rejuvenating” donor organs in the peri-transplant period so as to improve their tissue quality and promote tissue regeneration via specific antioxidant molecular signaling pathways [114].

Antioxidant molecular tools, like nanoparticles, must overcome inherent challenges before translational success is achieved. For example, biological barriers that prevent the intracellular delivery of antioxidants/anti-inflammatory therapeutics by inefficient endosomal escape will need to be overcome to enhance bioactivity at target sites. Reproducibility and scale-up synthesis remain formidable hurdles. Translating antioxidants or anti-inflammatory therapeutics into clinically viable products will require robust manufacturing pipelines, the standardization of surface modifications, and regulatory-compliant quality control. Another concern that must be considered is whether future antioxidants/anti-inflammatory therapeutics trigger immunogenicity or provoke unintended immune responses. The risk is heightened in hepatic IRI, where immune sensitivity is elevated. Moreover, the growing appreciation for differences in redox and immune states among patients needing liver transplantation will require personalized approaches that tailor treatments to individual oxidative stress profiles, immune status, or genetic predispositions. Finally, more studies need to focus on developing reliable biomarkers to measure the status of ROS, cytokines, or danger signals in real time. In this way, clinicians may use the dynamic modulation of feedback loops and better control immune–redox balance to manage hepatic IRI. Table 3 shows several representative clinical studies focused on therapeutic targets for ROS, collected from the years 2022–2025.

## 6. Conclusions

The interplay between oxidative stress, immune crosstalk, and feedback loops in hepatic IRI remains poorly understood. Understanding how these multiple feedback loops converge and influence each other may be key to identifying precise intervention points and improving outcomes in liver transplantation. Accumulating evidence suggests that managing ROS in the stressed liver may protect hepatocytes from damage and suppress immune activation through DAMP release and inflammatory signaling cascades. Integrating a systems biology approach that includes emerging antioxidants and immunomodulatory therapies could provide new avenues for personalized and more effective treatments.

## Figures and Tables

**Table 1:** Representative oxidative stress-related pathways and key findings from the period of 2024–2025.

Liver Disease	Gene/Pathway	Key Findings	Ref.
Iron Overload-Induced Liver Damage	TGF-β/Smad	Ellagic acid mitigates ferroptosis, alleviating liver damage.	[19]
Alcohol-Related Liver Disease (ALD)	TLR4/NF-κB/NLRP3	*H. pylori* FMT exacerbates ALD via LPS-induced pathway activation.	[20]
Liver Transplant-Related Biliary Injury	Nrf-2/HO-1, JNK	Nanomedicine inhibits ROS and modulates macrophages via Nrf-2/HO-1.	[21]
Hepatic Ischemia–Reperfusion Injury	ROS scavenging enzymes	Copper-based nanozymes scavenge ROS and protect liver.	[22]
Hepatic Ischemia–Reperfusion Injury	Keap1/Nrf2/HO-1	Crocetin preconditioning activates Nrf2/HO-1 by disrupting Keap1.	[23]
Fatty Liver and Ischemia–Reperfusion Injury	PI3K/AKT	Puerarin regulates PI3K/AKT to reduce liver IR injury.	[24]
Diabetic Liver Disease	RAGE, TGF-β, TNF-α	Crocin and losartan reduce fibrosis-related gene expression.	[25]
NASH	FXR	ROS-scavenging nanobubbles treat NASH via FXR activation.	[26]
NALFD	AMPK/ACC/SREBP1	Codonopsis polysaccharides reduce lipid accumulation and inflammation.	[27]
Alcoholic Liver Injury	NRF1-TFAM	Nobiletin preserves mitochondria via NRF1-TFAM signaling.	[28]
Alcoholic Steatosis	PCSK9	FAF2 silencing modulates PCSK9 to reduce steatosis.	[29]
Liver Fibrosis	miR-3667-3P/ACSL4	circ_0074763 regulates ACSL4 via miRNA axis in fibrosis.	[30]
CCl_4_-Induced Fibrosis	TGF-β1-Smad	Umbelliferone inhibits fibrosis through TGF-β1-Smad.	[31]
Chronic Liver Injury	Nrf2	COX-2 inhibition upregulates Nrf2 to reduce ferroptosis.	[32]
Hepatocarcinoma	PI3K/AKT, ROS	FDX1 downregulation activates mitophagy, promotes HCC.	[33]
Hepatocarcinoma	TRIM7/Nrf2	Fangchinoline inhibits HCC by regulating ROS via TRIM7/Nrf2.	[34]
Hepatocarcinoma	SHP2/PI3K	Hypoxia-induced ROS promotes HCC growth via SHP2/PI3K.	[35]
Hepatocarcinoma	HEXB	ROS induces EV release by up-regulating HEXB to promote HCC.	[36]

**Table 2:** Synthetic antioxidant enhancements to mitigate hepatic IRI.

Antioxidant	Structure/Category	Mechanism	Clinical Applications	Ref.
Targeted Delivery				
(PEG)ylation	Hydrophilic Polymer	Promotes particle circulation and therapeutic effects.	Use of PEGylated catalase (CAT-PEG) reduced hepatic IRI.	[97,98]
GalNAc	Amino Disaccharide	Binding with hepatocyte ASGPR enables nanoparticle targeting to liver cells.	Enhanced CRISPR gene editing therapy with specificity to the liver.	[109]
Mannose	Monosaccharide	Modifications taken up by Kupffer cells to reduce ROS.	Mannose-conjugated RIPK3 siRNA delivery improves treatment of atherosclerosis.	[99,110]
Aptamers	Single-stranded nucleic acid sequences	High-affinity aptamer sequences guide nanoparticles to desired location.	Aptamer conjugation to gold nanoparticles (AuNPs) as a targeted treatment for cancer.	[111,112,113]
Enzyme-Based				
Ultrasmall Cerium (CE)–Manganese Nanoclusters	Nanozyme	Displayed (SOD)- and (CAT)-mimetic activity promoted ROS scavenging activity.	Use of CE–Manganese nanoclusters reduced oxidative stress in a murine hepatic injury model.	[101]
Cerium Oxide	Metal Oxide	Reduces lipid peroxidation; increases catalase and GST activity; acts as a potent ROS scavenger.	Administration of cerium oxide via laparotomy prior to IRI improved outcomes in rat model.	[102]
Redox Signaling Modulators				
A-MPDA@Fe_3_O_4_@PVP	Polydopamine Nanodrug	Prevents Fe_3_O_4_ accumulation; mimics CAT and SOD activity; activates PPARγ/NF-κB pathway to reduce oxidative stress.	When administered under hepatic IRI conditions, promoted ROS scavenging, immunomodulation, and liver function.	[103]
RLLs + LY294002 and Oridonin	Liposomal Nanocarrier	LY294002 inhibits PI3Ks; Oridonin provides antioxidant and anti-inflammatory effects; RLLs enhance liver delivery via CD44 targeting and stabilize CYP450, reducing inflammation and injury.	The use of this combination treatment improved insulin sensitivity and lowered inflammation and fibrosis in NAFLD mouse.	[95]
Flavonoid Compounds	Polyphenol	Reduces hepatic IRI and OxS through nanoparticle scavenging and suppression of pro-inflammatory cytokines TNF-α, IL-1β, and IL-6.	Hydroxysafflor yellow A (HSYA) was shown to reduce intracellular ROS, apoptosis, DNA damage, and lipid peroxidation.	[108]

Abbreviations: Asialoglycoprotein Receptor (ASGPR), Catalase (CAT), Cluster of Differentiation 44 (CD44), 1,2-distearoyl-sn-glycero-3-phosphoethanolamine (DSPE), Ischemia–Reperfusion Injury (IRI), N-acetylgalactosamine (GalNAc), Nonalcoholic Fatty Liver Disease (NAFLD), Nuclear Factor Kappa B (NF- κB), Oxidative Stress (OxS), Peroxisome Proliferator-Activated Receptor Gamma (PPARγ), Phosphoinositide 3-Kinases (P13Ks), Polyethylene Glycol (PEG), Reactive Oxygen Species (ROS), Receptor-Interacting Protein Kinase 3 (RIPK3), ROS-responsive Liposomal Nanocarriers (RLLs), Small Interfering RNA (SiRNA), Superoxide Dismutase (SOD).

**Table 3:** Representative clinical studies focused on therapeutic targets for ROS, collected from years 2022–2025.

Study Focus	Therapy	Study Design Phase, N, Year	Novelty	Outcome	Ref.
Oxidative Stress					
ALD	CYP2EI inhibitor CMZ	RCT, P2, 60, 2023	Inhibition of ethanol metabolism pathway in decreasing liver toxicity and damage	CMZ treatment significantly decreased hepatic steatosis more effectively than no treatment or CZP treatment only.	[115]
Skin Damage	GSH synthesis booster GAP	RCT, 21, 2024	Increased stability of topical GSH protection through GAP formulation	Topical GAP application induced higher GSH expression in human keratinocytes and mitigated damaged-induced oxidative stress and ROS production.	[116]
Chronic Pain	Phytocannabinoid, omega-3 fatty acids, glucosinolates	RCT, 25, 2023	Decrease in ROS through dietary changes involving nutrients in hemp oil, calamari oil, and broccoli	Participants who received the multi-ingredient therapeutic supplement experienced significantly reduced ROS production and pain levels due to OxS.	[117]
Immune Cells					
Fanconi Anemia	Quercetin	CT, P1, 30, 2025	Treatment of immune-deficiency disorder through oral quercetin interventions	Some patients showed less ROS in blood and bone marrow stem cell regions, increase in platelet count, decrease in neutrophil and hemoglobin levels.	[118]
Myocardial Infarction	CoQ10	RCT, 131, 2024	CoQ10 as a therapeutic recovery agent post MI	MI patients who received CoQ10 experienced improved cardiac function recovery, and animal studies indicated decreased pro-inflammatory immune cell recruitment as the cause.	[119]
Cancer Immunotherapy	OX40 agonist GSK3174998	CT, P1, 138, 2023	Use of antibody GSK3174998 alone or in combination regimen with PD-1 blocker to inhibit cancer growth	While treatment was tolerated by patients, there was little success in inducing immune cell activation in controlling disease progression using either regimen.	[120]
IRI					
Aneurysm Repair	Sevoflurane and Desflurane	RCT, 80, 2024	Use of volatile anesthetics in avoiding OxS/IRI and ROS production during surgery	While individual biomarkers for OxS/antioxidant effect were not significantly different in treatment groups, there was an improved OXY-SCORE in patients who received Desflurane post aneurysm repair.	[121]
Myocardial Infarction	Pentoxifylline	RCT, P2, 161, 2023	Pentoxifylline intravenous pre-treatment ahead of PCI to avoid IRI	While treatment was tolerated by patients, there was no significant difference between treatment group and control.	[122]
Nerve cell recovery	miR-106a Nanoparticles carrying DEX	RCT, 2022	Use of combination treatment in nanoparticles to attenuate IRI in hippocampal neurons	Pro-inflammatory and apoptotic activity, as well as IRI reduced through treatment with miR-106a nanoparticles and DEX.	[123]

Abbreviations: Alcohol-associated Liver Disease (ALD), Clinical Trial (CT), Clomethiazole (CMZ), Clorazepate (CZP), Coenzyme Q10 (CoQ10), Cytochrome P450 2E1 (CYP2E1), Dexmedetomidine (DEX),Glutathione (GSH), Glutathione Amino Acid Precursor (GAP), Ischemia–Reperfusion Injury (IRI), MicroRNA-106a (miR-106a), Myocardial Infarction (MI), Oxidative Stress (OxS), Percutaneous Coronary Intervention (PCI), Phase (P), Programmed Cell-Death Protein 1 (PD-1), Randomized Controlled Trial (RCT), Reactive Oxygen Species (ROS).
